# Exploring the Efficacy and Duration of Therapeutic Effects of a Novel Iliohypogastric and Ilioinguinal Ablation Approach: A Stepwise Radiofrequency Ablation Technique

**DOI:** 10.7759/cureus.67843

**Published:** 2024-08-26

**Authors:** Helena Krogman, Bryan Stevens, Caitlin M Gray, Sanjeev Kumar

**Affiliations:** 1 Department of Anesthesiology, Division of Pain Medicine, University of Florida College of Medicine, Gainesville, USA; 2 Department of Pain Management, University of Florida College of Medicine, Gainesville, USA; 3 Department of Anesthesiology, North Florida South Georgia Veterans Health System, Gainesville, USA

**Keywords:** pain relief, interventional pain management, peripheral nerve ablation, chronic pain, iliohypogastric neuralgia, radiofrequency ablation

## Abstract

Radiofrequency ablation (RFA) targeting the iliohypogastric and ilioinguinal (IH/IL) nerves is a recognized treatment for lower abdominal neuropathic pain. Despite its effectiveness, RFA typically offers only a temporary reprieve, necessitating repeated procedures. RFA procedures of the IH/IL nerves have been well described but often result in patient non-compliance and commonly necessitate the use of increased sedation. This case report details an RFA technique as a novel therapeutic technique for managing neuropathic pain associated with IH and IL pain. The technique described was conducted on a patient with complex pain and profound hyperalgesia with remarkable patient compliance and, more importantly, with reduced sedation. This case report delves into the progressive interventions employed by this novel technique in a patient being treated for IH/IL neuralgia. This case report describes increased patient compliance and potentially increased safety profile associated with this innovative RFA technique in comparison to traditional RFA and steroid injection. The patient, whose pain was unresponsive to standard treatments, was thoroughly assessed and underwent multiple failed interventions requiring sedation before the novel RFA technique was considered. We describe the patient’s progression through various treatments, illustrating the benefits of this novel RFA method over the established ones. The discussion highlights the advantages of the new technique in terms of its effectiveness and the duration of its pain relief, offering valuable insights into the pain management field. This case contributes to the growing array of therapeutic strategies in pain medicine, potentially enhancing patient outcomes for those with IH/IL neuropathic pain.

## Introduction

Chronic pain from ilioinguinal (IL) and iliohypogastric (IH) neuralgia poses a persistent clinical dilemma, marked by the often unpredictable success and inherent patient discomfort of current treatment options. Radiofrequency ablation (RFA) has been well-established as an option for alleviating pain from IH/IL neuralgia through targeted nerve ablations. Providing a less invasive alternative to surgery while yielding superior outcomes compared to traditional steroid and anesthetic injections, this approach demonstrates promising results in the field of pain management [1]. This case study evaluates the safety and therapeutic efficacy of a novel RFA technique for IH and IL nerves, comparing its outcomes with those of traditional steroid injections and conventional RFA methods.

The impetus for this case report arises from the need to discern the differences between conventional IH and IL RFA methods and the novel technique, termed the “IH and IL stepwise RFA technique.” This stepwise technique aims to offer more effective pain management while decreasing the risks associated with the procedure. In brief, the modified technique utilizes an incrementally increasing RFA temperature over a set period of time and requires constant ultrasound visualization. By comparing the novel technique with steroid injections and traditional RFA, this research provides a relevant clinical context to evaluate its potential as a more tolerable and effective treatment alternative for patients, particularly those who cannot withstand the heat generated by standard RFA or those who have not found relief from conventional therapies [2,3]. 

## Case presentation

We present the case of a 43-year-old female patient with an extensive medical history, including asthma, bronchitis, breast cancer, diabetes mellitus, fibromyalgia, gastroesophageal reflux disease (GERD), and myocardial infarction. She sought evaluation for persistent chronic abdominal pain localized to the right upper/lower abdominal quadrants, right groin, and perineal area. The pain, described as throbbing, stabbing, and burning, had worsened following a recent robotic-assisted laparoscopic surgery to address a right inguinal seroma and recurrent hernia with mesh placement and adhesion lysis. The pain was determined to be neuropathic, following the distribution of the right IH and IL nerves, with her worst pain score of 9 of 10 and averaging 6 of 10. This pain significantly impacted her work, daily activities, and relationship with her husband and caused sleep disturbances and anxiety. Pain exacerbation was noted with physical activity and touch, while some relief was achieved with opioid medication, duloxetine (Cymbalta), and muscle relaxants. Previous treatments, including pregabalin (Lyrica) and gabapentin, were attempted but led to side effects.

It is important to note that the patient had a long history of chronic pain and several interventions that were largely ineffective in treating her pain adequately. The patient often required sedation while undergoing procedures that typically do not warrant sedation. She was able to undergo all procedures but often demonstrated extreme intolerance to needles associated with pain procedures. Her previous pain etiologies include sacroiliac pain, chronic lumbar radicular pain, chronic pelvic pain, right lower quadrant abdominal pain, thoracic spine pain, intercostal neuralgia, cervicalgia, and fibromyalgia. She received several interventions for each of these etiologies with varying degrees of efficacy but no permanent resolution of pain. The patient also had a spinal cord stimulator implanted, which was later removed due to concerns over pruritus, and later voiced regret after its removal due to the return of profound 10/10 lumbar radicular pain. She had aborted a cervical medial branch block due to intolerable pain and later was successful in completing the procedure. She underwent two subsequent RFAs in the cervical spine and the lower intercostal nerves in the lower thoracic region. Her sedation for the intercostal ablation required 2 mg of midazolam and 1000 µg of alfentanil, and the cervical medial branch ablation required 6 mg of midazolam and 3000 µg of alfentanil, which is much higher dosing than typically needed for these percutaneous procedures.

Returning to her described right lower abdominal pain, conservative management and physical therapy had failed, leading to an IH/IL nerve block with steroids amidst significant patient distress and yielding only six hours of pain relief. Subsequently, a novel IH/IL RFA approach was considered to improve procedural compliance and extend the therapeutic effect.

**Figure 1:**
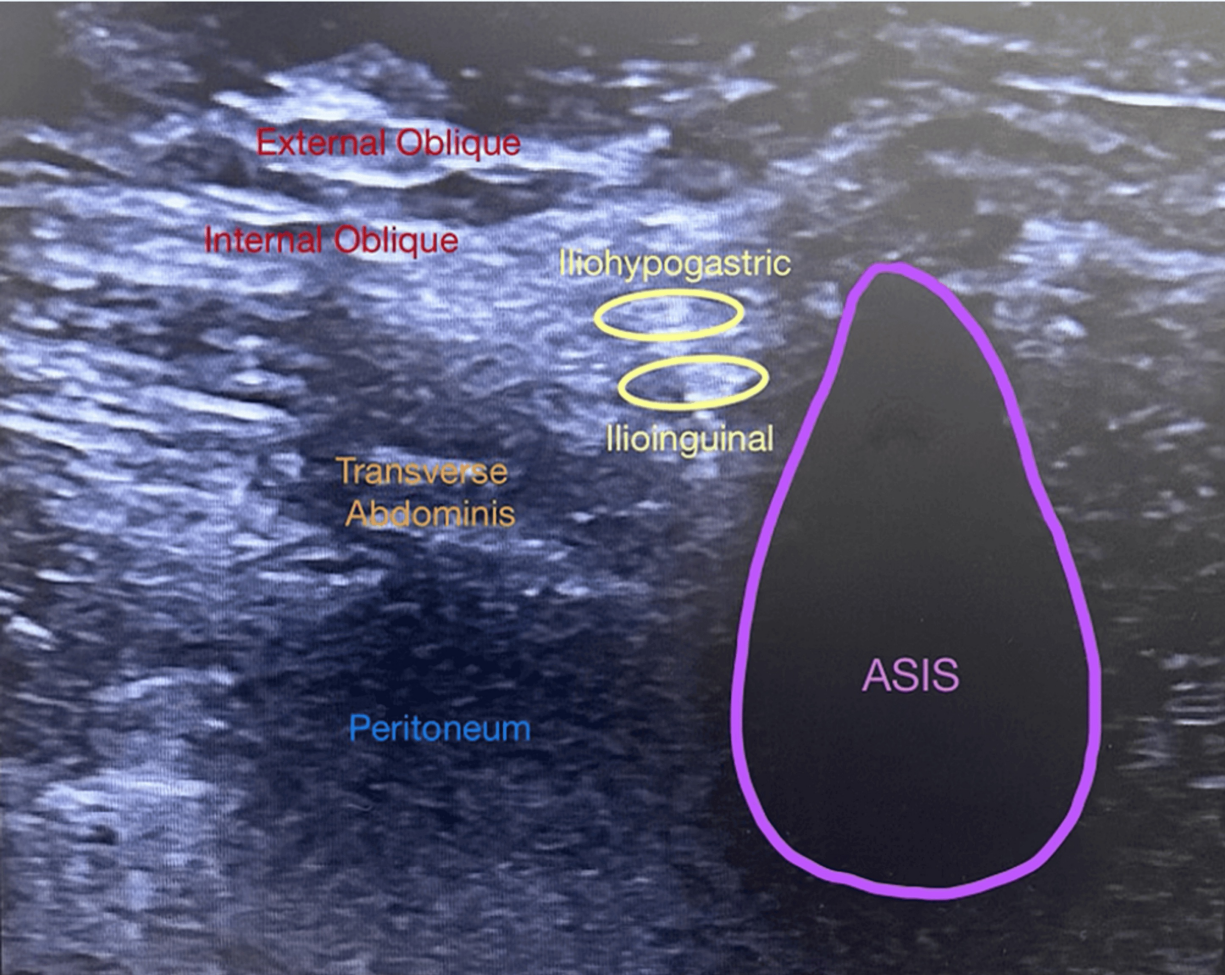
Ultrasound iliohypogastric and ilioinguinal procedural view *Note on intraoperative complications: During the procedure, potential complications may include but are not limited to bleeding, infection, accidental damage to surrounding structures, and transient procedural pain. Continuous and proficient ultrasound guidance and careful monitoring of patient responses are essential to minimize these risks.

Under ultrasound guidance with steady hands and minimal sedation, the patient’s IH/IL nerves were identified, and a 20-gauge RFA cannula was precisely positioned to be as close to the nerve/s as possible. Figure 1 highlights the procedural view of ultrasound-guided IH/IL nerves. A local anesthetic was not injected prior to ablation to maintain a good visual of the RFA cannula and the nerve/s and to identify the correct location of the nerve/s with sensory stimulation. Sensory stimulation at 50 Hz was commenced, and the patient reported concordant symptoms at 0.2 V indicative of the “intraneural” location of the RFA cannula. A stepwise RFA approach was used, starting at 50°C and in 5° increments up to 75°, with 60-second lesioning intervals. The patient remained comfortable and conscious throughout the six-minute procedure, reporting a significant reduction in right abdominal pain by the end. Ultrasound visualization confirmed that the hypoechoic lesion encompassed the targeted nerve locations. Pain scores improved from 7/10 to 2/10 post-procedure, with sedation limited to 2 mg of midazolam and 100 µg of fentanyl.

Despite the initial success, the patient did not experience long-term relief greater than three months and reported persistent perineal pain at her three-month follow-up, indicating a possible shift in pain localization rather than a treatment failure. It is important to note that this patient had a previous RFA using the traditional RFA technique with similar findings of recrudescence; however, the patient endorsed that the modified technique was far superior for intraoperative pain control. Considering her history of hyperalgesia and pain catastrophizing, her pain perception may be influenced by a generalized pain syndrome, with distinct pain generators sending impulses through the superior hypogastric plexus or the pudendal nerve, contributing to her ongoing lower abdominal/pelvic pain. A ganglion impar block with phenol was planned as a subsequent intervention at the time of this report.

## Discussion

In comparison to traditional RFA, this novel technique offers several distinct advantages, as exemplified in the presented case report and other bodies of literature by Lee et al. [4]. One notable improvement is the slow stepwise increase in temperature during the ablation process, contributing to increased patient compliance. This approach minimizes discomfort and allows the patient to remain conscious and comfortable throughout the procedure, with minimal sedation needed. Additionally, the real-time visualization of nerves and ablation under ultrasound guidance is a key element, eliminating the need for painful stimulation to confirm the location of nerves. This not only enhances the precision of the procedure but also reduces the risk of RFA early termination and subsequent neuritis. The combination of these advancements and the safety outcomes makes this technique a promising alternative to the classic method of IH/IL ablation [5,6].

**Figure 2:**
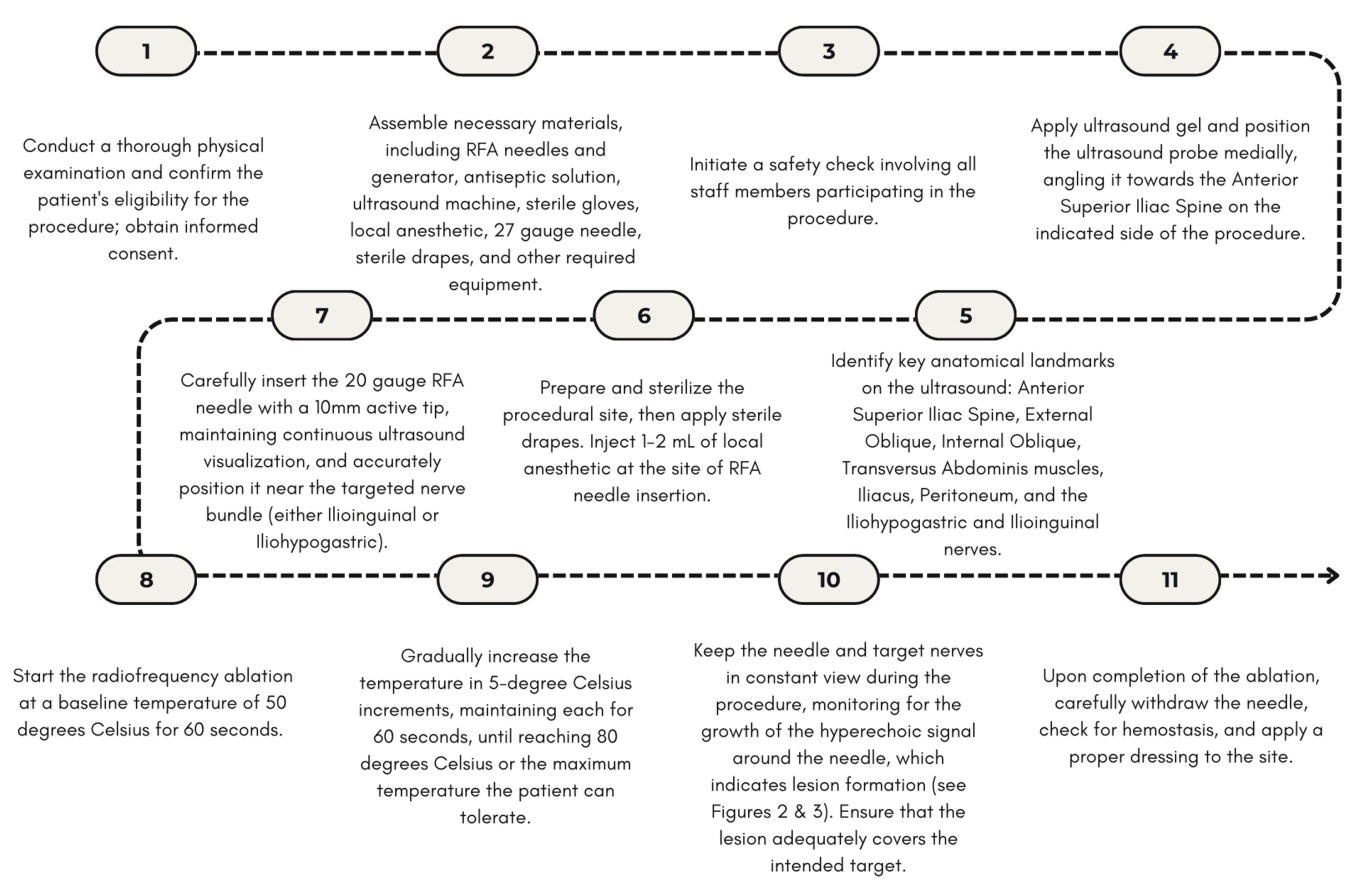
Stepwise radiofrequency ablation technique Image Credit: Helena Krogman This flowchart outlines the innovative stepwise radiofrequency ablation technique applied in this case study to target the iliohypogastric and ilioinguinal nerves. *Note on intraoperative complications: During the procedure, potential complications may include but are not limited to bleeding, infection, accidental damage to surrounding structures, and transient procedural pain [6]. Continuous and proficient ultrasound guidance and careful monitoring of patient responses are essential to minimize these risks.

The traditional RFA technique typically begins with utilizing ultrasound to identify the correct abdominal layer found between the internal oblique fascia and the rectus abdominis. It is often followed by sensory stimulation to confirm proximity to the target nerves, which often intensely provokes painful neuralgia. Next, after an injection of local anesthetic, a one-time ablation at 80° for a duration of between 90 and 120 seconds is performed. It is important to note that the traditional technique can adopt physician-dependent variability but largely follows this sequence of events. By contrast, the IH and IL stepwise RFA technique offers a more targeted and robust ablation that has methods that help mitigate painful RFA temperature pain and often negates the need for local anesthetic (see Figure 2). 

In this patient, the combination of the stepwise RFA temperature increases and persistent ultrasound guidance facilitated a successful ablation of the IH and IL nerves. This success is not only measured by the significant reduction in the patient’s pain intensity but also by the overall safety of the postoperative course. The transition from a preprocedural pain score of 7/10 to a post-procedural score of 2/10 underscores the effectiveness and safety of our approach to managing neuropathic pain. The safety profile for this procedure, as compared to her other ablation procedures, resulted in significantly less sedation. The stepwise technique resulted in a two-fold reduction in average midazolam use and a four- to eight-fold reduction in average opioid use when compared to previous RFAs. Her previous RFA procedures did, however, occur in other areas of the body, but the patient reported improved intraoperative tolerance, suggesting that the stepwise offers a less painful experience. Additionally, in cases where conservative management and previous interventions have failed, our technique offers a viable alternative for IH/IL pain intervention refractory to the more traditional techniques. However, a comprehensive patient evaluation is, of course, crucial to determine the suitability and efficacy in patients with treatment-resistant lower abdominal pain.

Despite the promising results of the stepwise technique, it is essential to acknowledge potential associated drawbacks. The use of incrementally increased temperatures during ablation may pose additional risks that are not yet known, and long-term effects are less described in the literature. Additionally, the need for specialized ultrasound guidance and minimal sedation may limit the applicability of this technique in certain patient populations who are unable to remain relatively motionless. Further research and clinical studies are warranted to assess the long-term safety and efficacy of our approach.

## Conclusions

In conclusion, the novel technique demonstrates promising safety and efficacy in the management of chronic abdominal pain caused by IH/IL neuralgia. The targeted visualization of the nerves and a stepwise, real-time visualization of the lesion increasing in size ensures more reliable therapy. Although this procedure requires total time under ablation, the patient can often tolerate this procedure due to the gradual increase in temperature and the ability of the patient to benefit from progressive lesioning and resultant analgesia. The aim of this case report is to detail a new technique that may be preferred in certain clinical scenarios, particularly in patients who poorly tolerate previous traditional ablation techniques. While further research is needed to validate its long-term efficacy and safety, this approach presents a viable option for individuals who have failed steroid injections and traditional ablation.
